# Re-Melting Behaviour and Wear Resistance of Vanadium Carbide Precipitating Cr_27.5_Co_14_Fe_22_Mo_22_Ni_11.65_V_2.85_ High Entropy Alloy

**DOI:** 10.3390/ma14081871

**Published:** 2021-04-09

**Authors:** Kai Treutler, Swenja Lorenz, Volker Wesling

**Affiliations:** Institute of Welding and Machining, Clausthal University of Technology, 38678 Clausthal-Zellerfeld, Germany; swenja.lorenz@tu-clausthal.de (S.L.); office@isaf.tu-clausthal.de (V.W.)

**Keywords:** high entropy alloy (HEA), wear, weldability, microstructure, ASTM G75, precipitating, vanadium carbide

## Abstract

High entropy alloys (HEAs) are among of the most promising new metal material groups. The achievable properties can exceed those of common alloys in different ways. Due to the mixture of five or more alloying elements, the variety of high entropy alloys is fairly huge. The presented work will focus on some first insights on the weldability and the wear behavior of vanadium carbide precipitation Cr_27.5_Co_14_Fe_22_Mo_22_Ni_11.65_V_2.85_ HEA. The weldability should always be addressed in an early stage of any alloy design to avoid welding-related problems afterwards. The cast Cr_27.5_Co_14_Fe_22_Mo_22_Ni_11.65_V_2.85_ HEA has been remelted using a TIG welding process and the resulting microstructure has been examined. The changes in the microstructure due to the remelting process showed little influence of the welding process and no welding-related problems like hot cracks have been observed. It will be shown that vanadium carbides or vanadium-rich phases precipitate after casting and remelting in a two phased HEA matrix. The hardness of the as cast alloy is 324HV0.2 and after remelting the hardness rises to 339HV0.2. The wear behavior can be considered as comparable to a Stellite 6 cobalt base alloy as determined in an ASTM G75 test. Overall, the basic HEA design is promising due to the precipitation of vanadium carbides and should be further investigated.

## 1. Introduction

Highly entropy alloys have been the subject of increasing scientific research in the last 20 years and are considered to be the next generation of materials in some areas because of their properties. Due to their composition of a larger number of different elements with approximately equal distribution as well as the possible addition of various other alloying elements, the number of potentially applicable alloying systems is immeasurable. Nevertheless, on the basis of various experiments and procedures, such as CALPHAD [1,2,3], it has been possible to identify and investigate promising systems for different applications. The fields of application range from high-temperature or cryogenic materials to corrosion-resistant and wear-resistant materials [4,5,6,7]. In addition to the single-phase high entropy alloys, other alloys have been developed which form precipitates in order to further influence the properties of these alloys [8]. Precipitated phases can be used to increase strength [9] and wear resistance, as in conventional non-high-entropy alloys [10]. One famous high entropy alloy is the so called Cantor alloy [11] which is investigated in various studies for example references [12,13] including its derivatives. This high entropy alloy and its derivatives consist of near equiatomic CrCoFeMnNi.

In addition to this alloy with its partly known properties, other systems are also being investigated for their properties. The results suggest a wide range of applications, ranging from cryogenic conditions [14,15] to high-temperature applications [16]. Furthermore, some of the high entropy alloys have very good corrosion resistance and wear protection properties [2,8]. Especially in wear protection, applied coatings of different materials are used, so that a change to high entropy alloys requires only minor adjustments on the process side.

The welding of high entropy alloys becomes more and more important due to the increased development in the field of alloy designs. This is reflected in the increasing number of publications in this field [17]. One aspect that is considered in the welding related HEA research so far are the mechanical and microstructural properties of the welds. Besides of the mechanical properties and the weldability the machinability becomes more important for HEA as well. Richter et al. showed that modern machining process have a beneficial influence on the surface integrity of a HEA [18].

The basic weldability of HEA is currently been seen as good with different welding processes [17,19,20]. Especially, for the CrCoFeMnNi basis the welding with laser or friction stir welding has been successfully performed [21,22]. The change in one alloying element in the concept can change its properties. So for each alloy the general welding technological occurring phenomena regarding the weldability and needs to be addressed besides metallurgical effects. These four main phenomena are:

- Hot-cracking
  Hot-cracking occurs due to shrinkage phenomena near the solidification of material and the lack of present melt to close the occurring gaps [23]. This phenomena is supported by the occurrence of a second low melting phase which solidifies in the interdendritic region of the first phase [23].
- Cold-cracking
  Cold cracking occurs due to high hardness and/or low toughness of the material [24]. This can be intensified due to external restraints or other phenomena like the hydrogen assisted cracking [25,26].
- Stress relief cracking
  Cracking which occurs due to high strength and low ductility during post weld heat treatment [27,28,29].
- Pore formation
  Pore formation occurs mostly due to a gap in the solubility of gases between the solid material and the melt [30].

For each of this phenomena there are designated welding test methods to verify the susceptibility to these phenomena. The basis for these tests is the remeltability of the material regarding the microstructural ability to form a suitable microstructure and that none of the weldability related phenomena occur without any additional constraints on the melt with for example free shrinkage.

In this study, the basic properties of a newly CrCoFeMoNiV alloy with small additions of Nb and Si is presented. This type of alloy exceeds in the studies on the wear resistance and the weldability/remeltability due to the exchange of Mn with Mo in the CrCoFeMnNi HEA and the addition of vanadium to the alloy to increase the wear resistance of the alloy by precipitating vanadium carbides. The possibility of precipitating hard phases for a similar alloy (CoCrFeNi_2.1_Nb_0.2_) have been shown by Sunkari et al., for Nb-containing hard phases [31]. The alloy used in this study can be called a complex concentrated alloy due to the intentional precipitation of carbides [2], but due to the fact that the matrix should be a single solution, the term high entropy alloy is used. The precipitation of carbides in similar HEAs has been reported by Huang et al., and the amount of carbides and the hardness of the alloy is directly linked to the carbon content [32]. The weldability and wear protection properties under abrasive wear are presented for CrCoFeMoNiV and are compared with conventional wear protection alloys such as Stellite 6 and Stellite 12. These materials are typically used in three-body abrasion, so the ASTM G75 “Miller Test” is used to characterize their wear properties. The wear resistance of these kinds of HEA are already of high interest and subject to studies in various alloy compositions [33,34,35,36]. The creation of an alloy-side basis for weldable wear-resistant high-entropy alloys is the aim of the investigations and the focus is on weldability. The integration of weldability into the alloy development represents a special approach that differs from the normally chosen path in alloy development, where the mechanical properties are in the foreground.

## 2. Materials and Methods

The methods used and the production of the alloy are described below. First the material and the casting process used will be described, secondly information on the applied wear test will be given followed by a description of the microstructural analysis methods and a short info about the used weldability test.

### 2.1. Used Material and Casting

The high entropy alloy was produced by casting small test pieces. A high-temperature furnace was used for this. The educts were therefore available in powder form in various master alloys. The mixing ratios of the different master alloys are shown in Table 1. These were mixed and melted in a graphite crucible at approx. 1600 °C under argon atmosphere. A total of 300 g was initially mixed and melted. The melt was kept at 1600 °C for 10 min under sequential stirring to ensure complete melting. The casting was done in a mould made of cast iron with the dimensions 60 mm × 30 mm × 10 mm. Cooling was done on air without any acceleration.

The targeted chemical composition of the alloy is shown in Table 2. The target alloy has an increased proportion of iron compared to the other alloy constituents. The proportion of carbon and vanadium should lead to the formation of vanadium carbide, which should precipitate in the matrix.

After casting, the surface was smoothed by grinding and samples were taken for metallographic examinations, welding and wear testing by wire electric discharge machining (EDM). These samples were freed from any erosion layer by grinding.

### 2.2. ASTM G75 Test

In order to map the three-body abrasive wear, the Miller test according to ASTM G75 is used [37]. This was originally developed to determine the wear in pipes that transport suspensions. With the help of the so-called Miller number, the abrasiveness of different suspensions can be determined and thus compared with each other [37,38,39]. For this purpose, [37] gives the chemical composition of a steel that is used for pumps in pipelines. Furthermore, the SAR number can be calculated with the Miller test. It is an indicator for the wear behaviour of a material in a certain suspension. If the suspension is kept constant and the material is changed, the materials can be compared in terms of their wear resistance using the SAR number. If the determined Miller number or SAR number is less than 50, the suspension is likely to cause only minor wear, while higher Miller numbers cause greater abrasive wear attack. With the help of these numbers, for example, the design of pumps can be carried out if the abrasiveness of the suspension used is known [10].

**Figure 1:**
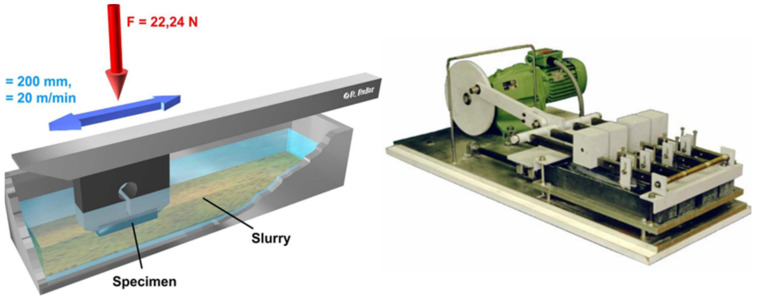
ASTM G75 (Miller Test) [10].

In this test, according to the standard, a sample is moved under a defined load of 22.24 N oscillating through an abrasive suspension against a counter body of chloroprene rubber at a speed of 20 m/min (Figure 1)).

At the end of each stroke, the sample is lifted so that there is abrasive material mixture in the gap between the sample and the counterbody at any time. The path length of one stroke is 200 mm. The total duration of the test is six hours, whereby the loss of mass of the samples is determined gravimetrically after every two hours [10].

**Figure 2:**
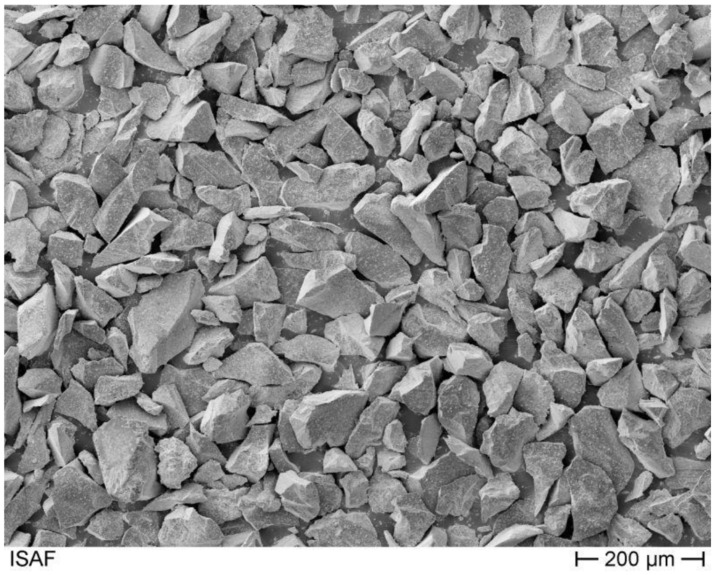
SEM Image of corundum F220 [10].

According to [37], the suspension used consists of 150 g solid and 150 g liquid. The standard material used for the tests at the Institute of Welding Technology and Separating Production Processes is a high-grade corundum of grain size F220 (d50 = 57.5 μm), (Figure 2) [10].

### 2.3. Micrstructural Analysis

For the metallographic analysis of the samples, they were embedded in a polymer and then automatically ground and polished. Subsequently, images were taken using an optical microscope and a scanning electron microscope. In order to emphasize the structure of the samples more clearly, they were etched. For this purpose, an etching solution according to Murakami with Murakami etchant (10 g KOH or NaOH, 10 g potassium ferricyanide, 100 mL water) was used. The preparation and evaluation of the micrographs has been conducted in accordance with the common standards including but not limited to: DIN CEN ISO/TR 16,060 “Etchants for macroscopic and microscopic examination” [40], DIN EN ISO 6520-1 “Classification of geometric imperfections in metallic materials” [41] and DIN EN ISO 17,639 “Macroscopic and microscopic examination of welds” [42].

### 2.4. Re-Melting Behaviour

In order to obtain a statement about the weldability at this stage of the alloy development, the cast samples were re-melted using a TIG welding process on one side of the sample. This then allowed statements to be made about the microstructure resulting from welding and welding-related problems such as hot or cold cracks and any pore formation to be identified.

## 3. Results

No casting-related issues arose during processing. The melt was sufficiently fluid and filled the mould well. A slag formed on the surface of the melt, but this was not examined more closely. Significant outgassing of the melt was also not observed. There were no cracks or shrinkage cavities. After grinding the specimen showed a metallic blank surface without any defects. Due to the forming slag an additional determination of the chemical composition has been done.

### 3.1. Chemical Composition

To determine the losses of alloying elements an EDX analysis has been carried out, Table 3. This showed that the intended increased iron content was reduced during melting and casting, Table 3. In addition, the amount of dissolved nickel was about 6 at.-% below the target. Correspondingly, the Cr and Mo contents are significantly higher. This suggests that the slag formed consists largely of iron and nickel.

Nevertheless, the microstructure exhibits the desired effects.

### 3.2. Microstructure

**Figure 3:**
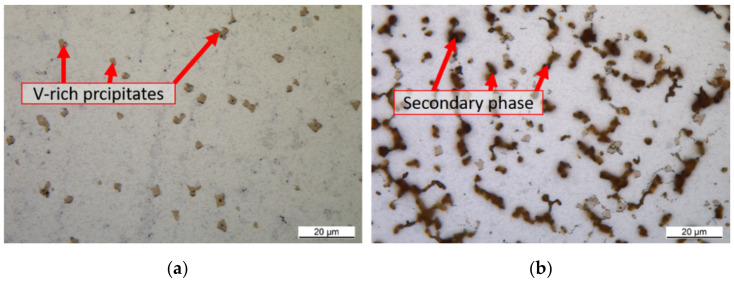
Microstructure of the as cast alloy (**a**) not etched, (**b**) Murakami solution.

Figure 3 shows the microstructure of the high entropy alloy not etched. Finely distributed yellowish precipitates with an average size between 1 µm and 2 µm are visible. The distribution is uniform over the entire cross-section. Next to the precipitates, two different phases are visible, Figure 3.

The secondary phase can be made visible by etching with Murakami solution (Figure 3). Here, the dendrites can be seen. This indicates that the second phase is a phase with reduced solidification temperature. Since the precipitates occur almost exclusively in the first phase, it can be concluded that they only form after solidification at lower temperatures.

**Figure 4:**
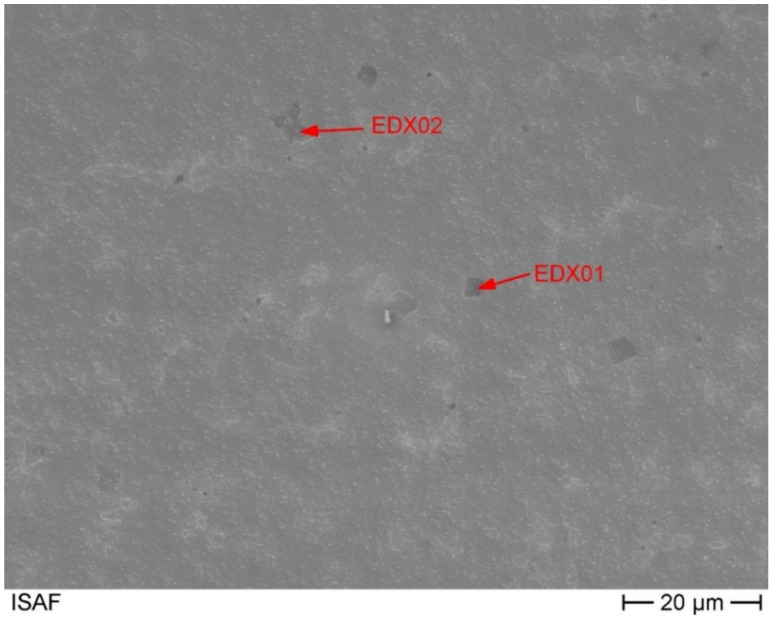
SEM image of the alloy with marks for EDX measurements of precipitates.

A detailed analysis of the chemical composition of the three different phases shows that the precipitates are vanadium, chromium and nickel rich phases (Figure 4 and Table 4). In addition, significant amounts of cobalt and iron are present as well. Due to the large difference in the carbon content, it can be stated that this alloy has two different precipitates forming. One is a chromium-vanadium carbide and one is a chromium-vanadium intermetallic.

**Figure 5:**
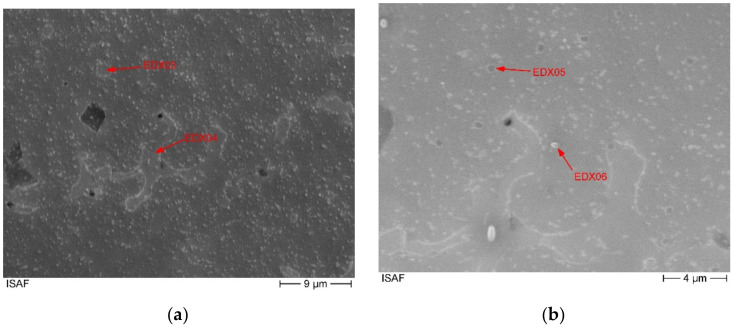
SEM images of different magnifications (**a**) 2500×, (**b**) 5000× of the alloy with marks for EDX measurements of two main phases.

**Figure A1:**
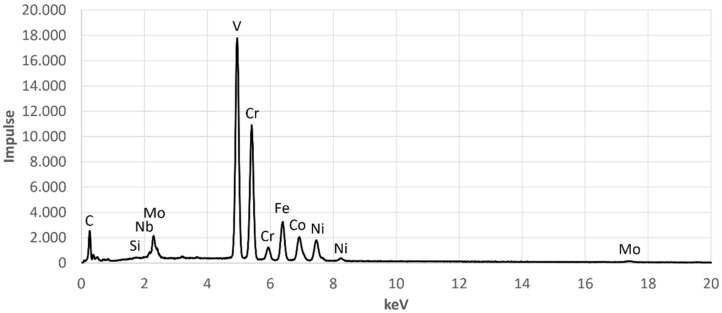
EDX-Spectrum for EDX Point 1.

**Figure A2:**
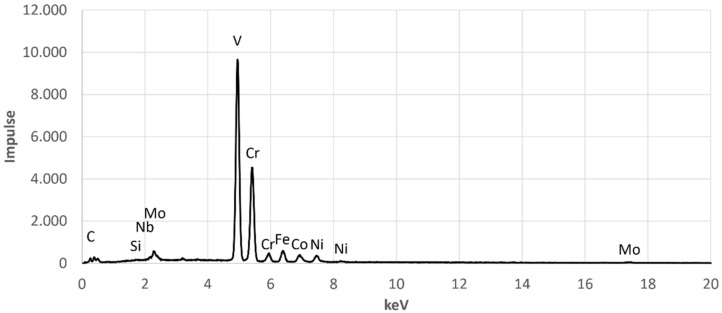
EDX-Spectrum for EDX Point 2.

The corresponding spectra of the presented measurements are given in Figure A1 and Figure A2, which can be found in the Appendix A. The chemical composition of the second phase, measured by EDX, is shown in Table 5. The corresponding measuring points are marked in the SEM image (Figure 5). The second phase of the matrix shows a reduced molybdenum content compared to the surface measurement of 6 at.-% and 12 at.-% with simultaneously high iron, cobalt and especially nickel contents. This corresponds to an average reduction of the molybdenum content of 13 at.-%. The nickel content in this phase is twice as high as in the entire alloy. The chromium content is missing by 7 at.-% and the iron content is increased by 6 at.-%. This, in conjunction with the area analysis to determine the overall chemical composition, shows that the main phase is a molybdenum-rich phase and the second phase is a molybdenum-poor phase. The increased nickel content in the second phase leads to the assumption that the solubility of nickel in the molybdenum rich matrix alloy is limited. And vice versa that there is a corresponding alloy nickel rich alloy composition with limited molybdenum solubility. Incomplete melting can be dismissed due to the clear presence of chromium cobalt and iron. In addition, the larger areas of the second phase no longer have a particle-like shape.

**Figure A3:**
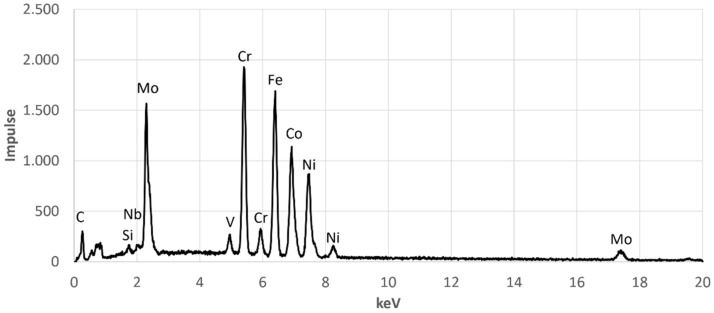
EDX-Spectrum for EDX Point 3.

**Figure A4:**
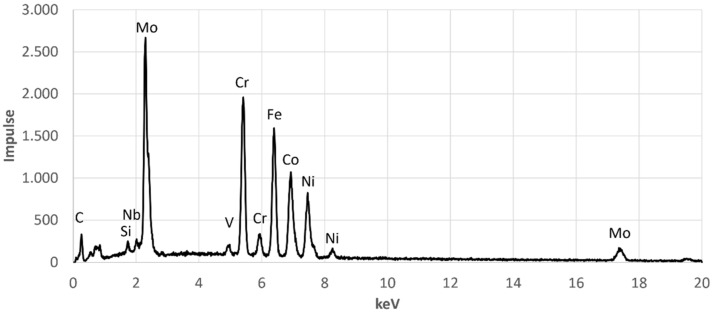
EDX-Spectrum for EDX Point 4.

Furthermore, Figure 5 shows that over the entire cross-sectional area there are further precipitates with a size of a few hundred nanometres. The composition of these fine precipitates indicates that they are not precipitates but the second molybdenum-poor phase, Table 5. The corresponding EDX spectra are shown in the Appendix A in Figure A1, Figure A2, Figure A3 and Figure A4.

The formation of two phases is rather unfavourable for a possible weldability of the alloy, since two-phase systems with a dendritic growth as shown in Figure 4 and a low-melting phase within the interdendritic space have a greater risk of hot cracking, [23]. However, there are approaches to modify the original alloy design to a single-phase system. For this purpose, the molybdenum content could be adjusted. Detailed investigations are needed here.

### 3.3. Remelting Behaviour

**Figure 6:**
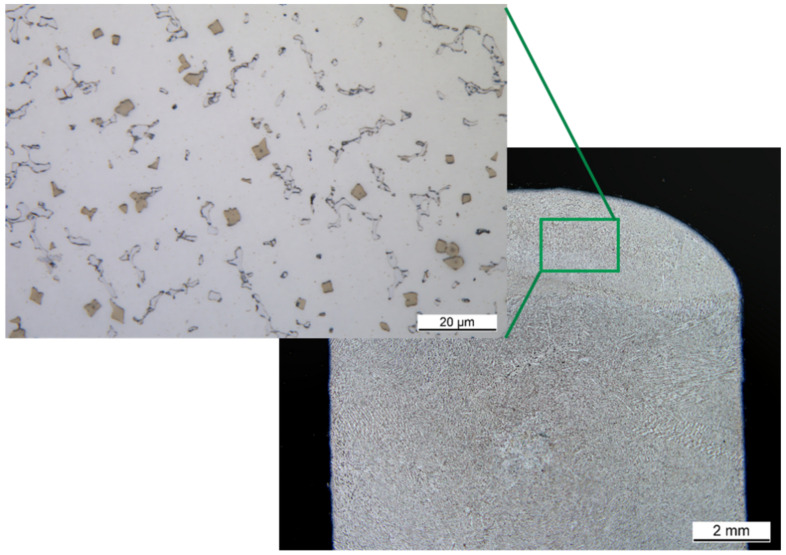
Microstructure of the re-melted sample.

In order to be able to give a basic assessment of the weldability of the alloy, the samples were locally remelted with the help of a TIG welding process with argon as shielding gas and examined for the resulting microstructure. A current of 100 A with a tip to workpiece distance of 10 mm has been used. The specimen was 10 mm × 10 mm × 50 mm and was melted on the smaller surface at on end. The heating/melting was done until the full face of the specimen was molten. Figure 6 shows the microstructure of the remelted area.

The lower right part of Figure 6 shows an overview of the remelted area and the base material. As can be seen, there is no distinct heat-affected zone and no clear difference in precipitation distribution between the re-melted area and the base material. Neither hot cracks nor pore formation were observed. At most, the remelted area is slightly finer in terms of its phase distribution and size. This is due to the accelerated cooling during welding compared to the casting tests. In general, the accelerated cooling and the change in the distribution of the phases should result in a change in the hardness of the material. Overall, it can be stated that the used high entropy alloy has a basic remeltability.

### 3.4. Hardness

**Figure 7:**
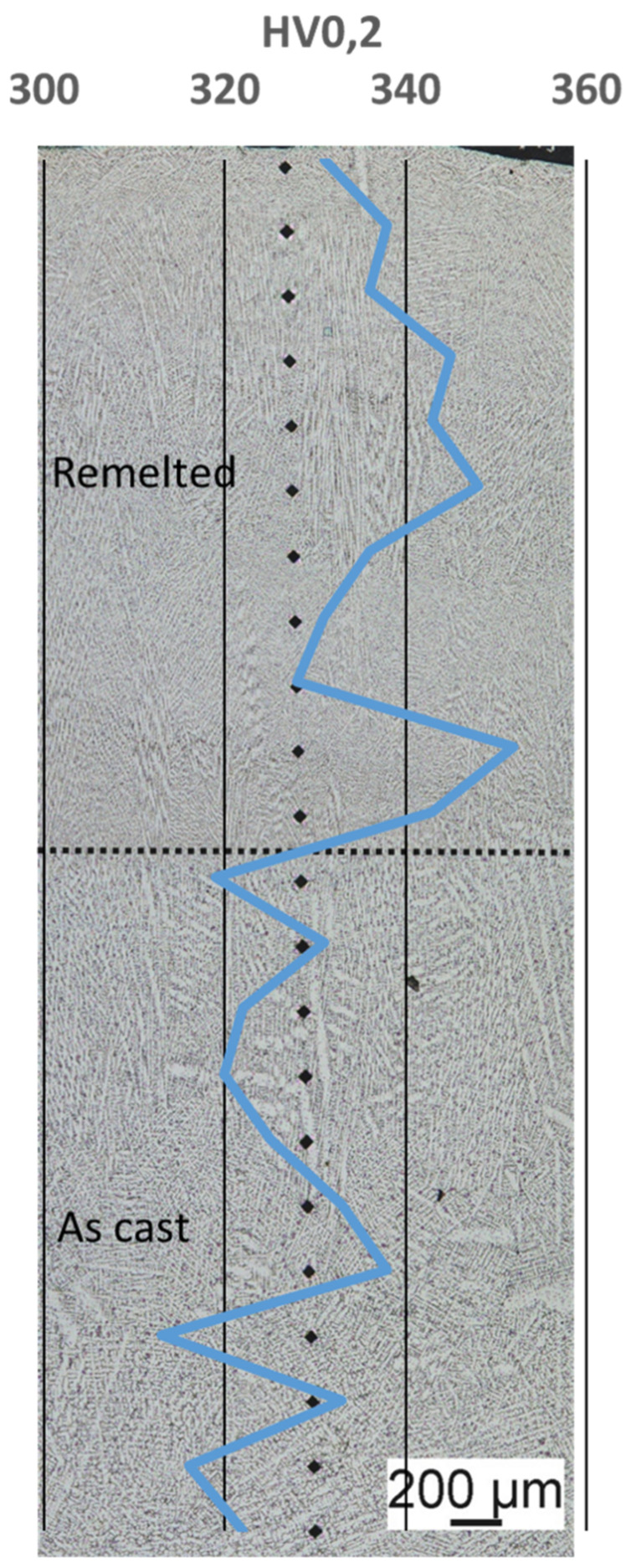
Hardnessprofile of re-melted alloy and base material.

The hardness measurement across the cross-section of the sample showed that the base material has a hardness of 324HV0.2 and the remelted area has a hardness of 339HV0.2 on average, Table 6. The series measurement carried out to identify different areas also below the re-melted material is shown in Figure 7. The variations that occur within the measurements are within the normal range for hardness measurements on welded and materials. The hardness measurements also show that there is no distinct heat-affected zone with changed properties, as is typical for other material groups, like high strength low alloyed steels. Due to the considerable high hardness of the material a good wear resistance under three body abrasive wear conditions can be anticipated.

### 3.5. Waer Resitance

In order to be able to compare the properties of the high-entropy alloy used with other wear protection materials used, the wear resistance of cladded Stellite 6 and Stellite 12 was determined. Stellites are hard alloys based on cobalt-chromium [10].

**Figure 8:**
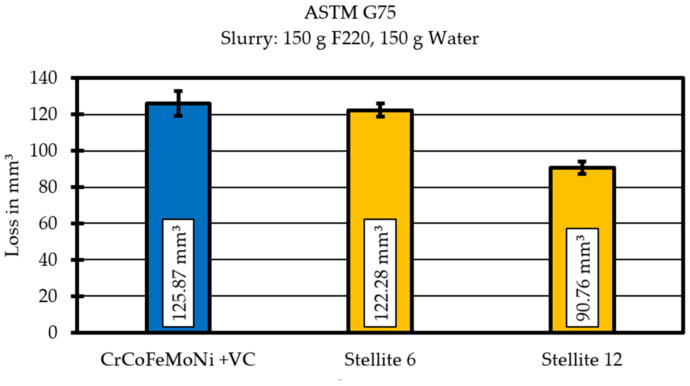
Mass loss for CrCoFeMoNi + VC and Stellite 6 and Stellite 12.

These two alloys are currently widely used as protection in abrasive three-body wear. In the ASTM G57 “Miller Test”, these show an ablation of 91 mm^3^ for the Stellite 12 and 122 mm^3^ for the Stellite 6. In comparison, the CrCoFeMoNi + VC high entropy alloy used shows an ablation of 126 mm^3^ (Figure 8).

These results show that the wear resistance of the CrCoFeMoNi + VC alloy is within the range of conventionally used wear protection alloys and clearly shows potential for a further increase in wear resistance by, for example, increasing the proportion of carbides by increasing the carbon content of the starting alloy.

## 4. Discussion and Summary

Due to the possible casting of the material the presented alloy is in line with comparable results from the literature for casted or cladded CrCoFe-containing HEAs [43]. The intended chemical composition could not be reached through casting and some alloying elements were lost in a slag. In the as-cast materials two phases besides precipitates are present. One a molybdenum rich and one a molybdenum poor and nickel ridge phase. A similar phase distribution has been reported for an AlCrFeNiMo HEA produced by laser-cladding and there a similar reduction of molybdenum in the second phase has been reported [43]. Furthermore, the microstructure contains vanadium-rich precipitates, carbides and another type of precipitation. The possibility of precipitating other phases during a heat treatment has been reported for Cu-rich phases in [9,32]. Vanadium-rich precipitates also occur after a conducted remelting as well as the two main phases. A specific analysis of the precipitated phases and the main phases with regard to their atomic structure and composition has not yet been carried out, but will be aimed at after further adaptation of the alloy system. The occurrence of a secondary matrix phase in the selected alloy system can lead to the formation of hot cracks during welding processes. The formation of hot cracks through different phases is a known phenomenon for many material systems [23]. However, the results presented for the remelting tests show no evidence of such behaviour without external restraints, It should be noted, however, that these effects usually occur more frequently when external residual stress-inducing restrictions, such as restraints or distortion, are present. The same applies to the consideration of cold cracks or stress relief cracking. These mostly occur in large components with the potential for the formation of high residual stresses and in connection with embrittlement of the material. In future work adequate welding test for determining the cold cracking susceptibility have to be applied, which are well summarized by Kannengiesser et al., in [44]. The found absence of an pronounced HAZ is reported in the literature for AlCrFeNiMo and low molybdenum contents as well [43]. The modification of the presented alloy design can lead to a pronounced HAZ, which is common for other HEA steels and aluminium alloys.

The results for the hardness (of 324HV0.2) of the high entropy alloy are in line with some similar alloys without molybdenum from the literature [32] and furthermore show the influence of the cooling time on the hardness. A faster cooling increases the hardness to 339HV0.2. Here, an approach for the modification of the presented alloy-design can be derived from. The increase of the carbon content should increase the hardness of the material and, hopefully, increase the wear resistance against three body abrasive wear. The effect of the carbon content on the precipitation behaviour in HEA has been demonstrated by Huang et al. and can be transferred [32]. Furthermore, it has been shown that the addition of other alloying elements can have a significant influence on the phase morphology, the hardness and the wear morphology. The addition of small amounts of Titanium may have a positive effect like shown by Löbe et al. [35].

Nevertheless, it should be noted that the methods used for determination of the chemical composition, the EDX measurements, suffer from some restrictions in their accuracy. The measured values have been reported as given in the measurement results.

The wear test results the show that the chosen alloy design can compete with commonly used hardfacings like Stellite 6.

Overall, it can be stated that the results presented herein are in line with other HEA studies and the usage of Mo as alloying element in CrCoFeNi-based alloy systems is possible. This type of alloy can be further strengthened by the addition of vanadium due to the precipitation of vanadium-rich phases.

## 5. Conclusions

The presented study shows that the used CrCoFeMoNi + VC high entropy alloy has the following features:

- Castable

The small samples produced showed no casting defects and a homogeneous microstructural morphology. The microstructure present in the as cast specimen show precipitations and two common main phases. One molybdenum rich and one molybdenum poor phase.

- Contains vanadium carbide precipitates

As intended, precipitates were found in the microstructure of the high entropy alloy, which have an increased vanadium and carbon content.

- Hardness and heat affected zone

The presented alloy with its vanadium rich precipitated reaches a hardness of 324HV0.2. The TIG-remelted material has a slightly higher hardness of 339HV0.2. A pronounced HAZ with an increase or drop in the hardness has not been observed. The microstructure shows no evidence for a pronounced HAZ as well.

- Remeltable

The remeltability investigations carried out show no indications of welding-related failures which need to be supported with corresponding welding tests as there is a possibility of hot cracking due to the second phase in the matrix of this alloy. Pore formation or cold cracks could not be found during and after remelting.

- Wear resistant

Under three-body abrasion wear testing in the G75 “Miller test” the alloy showed similar behaviour to conventional cobalt-chromium hard (Stellite 6 and Stellite 12) alloys.

Furthermore, it can be stated that the given Cr_27.5_Co_14_Fe_22_Mo_22_Ni_11.65_V_2.8_ high entropy alloy show the potential of generating a remeltable wear resistant alloy, but further work on the alloy design and the determination of its properties is necessary. Especially, the dependence of the precipitation behaviour from the carbon content is of great interest for this promising new type of alloy.

## Figures and Tables

**Table 1:** Used pre alloys for casting.

Weight-%							
FeNb	FeMo	Fe	FeSi	FeV	Ni	Cr	Co
0.14	24.4	19.25	0.24	3,47	17.5	17.5	17.5

**Table 2:** Intended chemical composition of the used HEA in advance of casting.

Element in at.-%								
Fe	C/B	SI	Cr	Mo	Co	V	Nb	Ni
25.65	0.3	0.5	17.5	18.1	17.5	2.85	0.1	17.5

**Table 3:** Chemical composition of the used HEA in advance of casting (EDX).

Element in at.-%					
Fe	Cr	Mo	Co	V	Ni
22.21	27.51	22.06	13.82	2.85	11.66

**Table 4:** Chemical composition of the precipitates (EDX).

Position	Element in at.-%						
-	Fe	Cr	Mo	Co	V	Ni	C
EDX1	11.68	22.63	1.59	8.41	38.89	9.39	7.13
EDX2	5.57	25.88	0.89	3.57	58.14	5.02	0.7

**Table 5:** Chemical composition of the used HEA in advance of casting (EDX).

Position	Element in at.-%					
	Fe	Cr	Mo	Co	V	Ni
EDX3	27.55	22.19	6.64	20.91	1.7	21.77
EDX4	26.23	18.75	12.32	19.64	1.03	18.39
EDX5	30.27	17.99	2.97	21.43	1.13	26.21
EDX6	28.90	18.42	3.8	21.31	1.09	26.24

**Table 6:** Hardness of the used alloy.

Material Condition	Hardness HV0.2	Deviation
As Cast	324	7.2
Re-melted	339	7.3

## Data Availability

The used data is available on request from the corresponding author.
